# Key components of the mental capacity assessment of patients with anorexia nervosa: a study of three countries

**DOI:** 10.1186/s40337-022-00633-7

**Published:** 2022-07-26

**Authors:** Yoshiyuki Takimoto

**Affiliations:** 1 Department of Biomedical Ethics, Faculty of Medicine, The University of Tokyo, Tokyo, Japan; 2 Department of Psychosomatic Medicine and Stress Science, Faculty of Medicine, The University of Tokyo, Tokyo, Japan

**Keywords:** Anorexia nervosa, Treatment refusal, Clinical ethics, Mental capacity, Decision-making

## Abstract

**Background:**

Patients with anorexia nervosa (AN) often refuse treatment despite their extremely low nutritional status. This study investigated the methods of assessing the mental capacity of patients with anorexia nervosa (AN) who refuse treatment by physicians in Japan, the United Kingdom (UK), and the United States (USA). It also identified the key points of the assessment.

**Methods:**

A questionnaire survey using a case vignette was conducted among physicians (Japan, n = 53; UK, n = 85; USA, n = 85) who treat eating disorders.

**Results:**

A total of 23% of physicians in Japan, 32% in the UK, and 35% in the USA reported that they believe patients with AN lack the capacity to make appropriate decisions. Physicians who considered patients with AN to have an impaired mental capacity placed significantly more emphasis on the level of psychopathological values, which are values caused by AN (and can be changed by recovery) that affect the ability to be rational, when assessing the mental capacity of these patients. Conversely, physicians who considered patients with AN to have full mental capacity placed significantly more weight on the ability to express a choice or preference.

**Conclusions:**

It may be necessary to add the level of psychopathological values to the assessment of the mental capacity in relation to obesity fears and emotional disturbances of Patients with AN because emotions caused by psychopathological values strongly influence decision-making. By considering the level of psychopathological values, it may be feasible to reflect the actual situation during the assessment of the mental capacity of those who refuse AN treatment, thus making it more likely to overcome ethical dilemmas.

**Supplementary Information:**

The online version contains supplementary material available at 10.1186/s40337-022-00633-7.

## Plain English summary

This study investigated the method of assessing the mental capacity of anorexia nervosa (AN) patients who refuse treatment in Japan, the United Kingdom, and the United States. Approximately one-third of clinicians who assessed patients with AN declared that those patients showed impaired decision-making capacity when refusing treatment. Clinicians who considered patients with AN to have impaired decision-making ability tended to focus on the level of psychopathological values when assessing their mental capacity.

**Supplementary Information:**

The online version contains supplementary material available at 10.1186/s40337-022-00633-7.

## Background

Anorexia nervosa (AN) patients often refuse treatment despite their extremely low nutritional status, which requires immediate treatment. However, informed consent must be obtained from the patient before performing therapeutic actions. Therefore, treatment refusal by patients with AN causes an ethical dilemma for physicians. For example, if patients refuse treatment when it is necessary, then prioritization of the protection of life by physicians can infringe on the self-determination of these patients. Legal disputes and ethical debates regarding whether coercive treatment should be administered to patients with AN who refuse treatment have occurred [1].

One of the important keys to assessing patients with AN who refuse treatment is determining whether the patient has the mental capacity to make appropriate decisions. Regarding the ethics of coercive treatment in the field of psychiatry, coercive treatment can be justified only when the patient's capacity to provide consent is impaired and severe danger to health or life cannot be prevented by less intrusive means [2]. If patients with AN who refuse treatment have sufficient mental capacity, then, from an ethical perspective, it is necessary to respect their refusal of treatment as an autonomous decision. Therefore, it is essential to assess the mental capacity of patients with AN when they refuse treatment.

Mental capacity is generally evaluated according to four elements: understanding; appreciation; reasoning; and expression [3]. An assessment of the mental capacity to consent to treatment is usually performed by the treating clinician when the mental capacity of the patient is unclear. However, this clinical assessment is known to overestimate the mental capacity of patients [4].

Often, patients with AN may not demonstrate obvious impairment in these four factors, even when they refuse treatment. However, it is impossible to consider that patients with AN have impaired mental capacity without finding impairment in terms of the four aforementioned factors. The MacArthur Competence Assessment Tool for Treatment (MacCAT-T) is one of the gold standards for assessing the mental capacity [5]. A small qualitative study [6] using the MacCAT-T did not show any problems with the mental capacity to consent to treatment for a sample of 10 adolescents with AN who had been severely ill. However, a quantitative study of 35 adolescents with AN [7] showed that they had mild problems with reasoning compared to healthy controls. Elzakkers et al. examined the mental capacity of patients with AN based on the judgement of the clinicians and the MacCAT-T results [8]; 29% of patients with full mental capacity according to the MacCAT-T were considered to have diminished mental capacity based on clinical judgement, and 48% of patients who were considered to have diminished mental capacity based on clinical judgment had full mental capacity according to the MacCAT-T. Therefore, the consistency between clinical judgement and MacCAT-T scores is not high.

For those with AN, the assessment of mental capacity using the MacCA-T has been noted to focus heavily on cognitive function and focus little on the values of the patients [6, 9–12]. The results of previous studies indicated that during the assessment of the mental capacity of Patients with AN, there are factors that are difficult to be captured by the four aforementioned elements [3]. Although previous studies have identified the clinical assessment as one criterion for evaluating the mental capacity, they have not investigated what experienced clinicians focus on when assessing the mental capacity other than its general components. Therefore, factors other than understanding, appreciation, reasoning, and expression for assessing the mental capacity of patients with AN who refuse treatment should be classified to help reduce variability among clinicians performing these assessments. However, no studies have investigated what physicians value when assessing the mental capacity of patients with AN who refuse treatment. Therefore, this study aimed to determine how physicians assess the mental capacity of patients with AN by conducting a survey among eating disorder therapists in Japan, the United States (USA), and the United Kingdom (UK). It also identified the key points of the mental capacity assessment.

## Methods

A case involving a patient with AN who refused treatment was developed, and a questionnaire survey was conducted to assess the mental capacity of that patient (Additional file 1: Appendix 1). The questionnaire required the physicians to evaluate whether the patient had any ability to make decisions, whether the wishes of the patient were respected, whether the patient had no ability to make decisions, and the reasons why the physicians determined their decisions. Additionally, the respondents were provided 10 questions with a multiple-choice format to indicate what they consider important when evaluating the mental capacity of patients with AN (Additional file 1: Appendix 1). The 10 questions were related to decision-making or cognitive ability [3, 13, 14].

We conducted an anonymous self-administered questionnaire survey delivered by mail to 212 members of the Japanese Society for Eating Disorders. For comparison, an anonymous web-based questionnaire with similar questions created and validated by back-translation was conducted among eating disorders specialists in the USA and UK. In the USA, a web-based survey was conducted among physicians who were registered in MDLinx (> 415,000 physicians) as members of eating disorder-related societies, such as the Academy of Eating Disorders, and those who treated eating disorders. In the UK, a web-based survey was conducted among physicians registered in Doctors.net.uk (> 200,000 physicians) who are members of eating disorder-related societies, such as the British Eating Disorder Academy, and who treated eating disorders. The web survey was conducted through a survey company that solicited responses until more than 80 responses were collected, assuming that the maximum response rate in Japan was 40%. In both the USA and the UK, three announcements encouraging participant in the survey were provided over a 6-week period.

### Statistical analysis

A chi-squared test was conducted to determine the significant difference in the proportion of responses among the three countries regarding the presence of mental capacity, reasons for the lack of mental capacity, and respect for self-determination. When significant differences were found among the three countries, a chi-squared test and Bonferroni's correction were conducted. For the items that were important for assessing the judgment ability, Fisher’s direct method was used to examine the differences in the response rates. All analyses were two-tailed, and *p* < 0.05 was considered statistically significant.

## Results

### General characteristics of the respondents

Fifty-three responses were obtained from physicians in Japan who specialize in treating eating disorders (25% response rate). There were 21 psychosomatic physicians, 25 psychiatrists, and 7 adolescent medicine physicians. Psychosomatic physicians were trained in the field of internal medicine and received additional psychiatric-psychosomatic training. Both psychosomatic physicians and psychiatrists mainly treat eating disorders in Japan. Most physicians had 10 to 19 years of experience, but some had more than 30 years of experience. Most physicians treated between 50 and 99 patients per year, whereas some treated up to 199 (Table 1).

**Table 1:** Characteristics of subjects

	Years of experience as a clinician				
	< 5 years	5–9 years	10–19 years	20–29 years	> 30 years
Japan (n = 53)	0	8	19	12	14
UK (n = 85)	2	8	50	19	6
US (n = 85)	4	17	32	21	5

	Number of AN patients examined in a year						
	< 20 patients	20–49 patients	50–99 patient	100–149 patients	150–199 patients	200–299 patients	> 300 patients
Japan (n = 53)	0	8	19	11	15	0	0
UK (n = 85)	0	46	22	8	2	3	4
US (n = 85)	0	0	42	24	4	8	7

*UK* United Kingdom, *US* United States of America, *AN* anorexia nervosa

Eighty-five responses were obtained from the UK. All respondents were psychiatrists. Of the physicians who responded, 28.2% worked in clinics that specialized in treating eating disorders, 24.7% worked in hospitals that specialized in treating eating disorders, and 57.0% worked in other medical facilities. Most physicians had 10–19 years of experience, whereas some had 20–29 years of experience. Most physicians treated 20–49 patients per year for eating disorders, and some treated between 50 and 99 per year.

Eighty-five responses were obtained from the USA. All respondents were psychiatrists; 44.7% worked in clinics that specialized in treating eating disorders, 16.5% worked in hospitals that specialized in treating eating disorders, and 38.8% worked in other medical facilities. Most physicians had 10–19 years of experience, and some had 20–29 years of experience. Most physicians treated between 50 and 99 patients per year, and some treated between 100 and 149 patients per year.

The total sample size for the three groups required for statistical analysis was 90. This value was calculated in accordance with previous studies by setting the difference at 40 points [3] (α = 0.05 and β = 0.1).

### Assessment of the existence of mental capacity

Approximately 70% of physicians considered the patient described in the questionnaire as partially impaired but capable of making decisions. However, 23% of physicians in Japan, 32% of physicians in the UK, and 35% of physicians in the USA considered the patient to have no mental capacity (no significant difference; *p* = 0.44; χ^2^ = 3.757) (Table 2).

**Table 2:** Mental capacity assessment

	Full mental capacity	Diminished mental capacity	Lack of mental capacity
Japan (n = 53)	6	35	12
UK (n = 85)	12	46	27
USA (n = 85)	13	42	30

*p* = 0.44. Χ^2^ = 3.757. df = 4

### Reasons for assessing the lack of mental capacity

Psychopathology was the most common reason provided by physicians who judged the patient to be incompetent to make decisions; 58% of physicians in Japan, 90.0% of physicians in the USA, and 52% of physicians in the UK provided psychopathology as the reason. Compared to physicians in other countries, a higher percentage of physicians in the USA provided this reason (no statistically significant differences; vs. Japan, *p* = 0.07; vs. UK, *p* = 0.09; Fisher’s exact test) (Table 3).

**Table 3:** Reasons for the lack of mental capacity

	Decreased level of consciousness because of malnutrition	Psychopathology of AN	Other reason
Japan (n = 12)	4	7	1
UK (n = 27)	8	14	5
USA (n = 30)	1	27	2

*p* = 0.018. Χ^2^ = 11.926. df = 4

### Respect for self-determination

Respondents who answered that the patient had not lost mental capacity were asked whether they would respect this patient’s self-determination to refuse treatment. In the USA and the UK, 67% and 44% of physicians, respectively, responded that they would respect it, whereas only 18% of physicians in Japan agreed that they would; this was a significantly lower percentage compared to that of physicians in the USA and the UK (Table 4).

**Table 4:** Respect for self-determination

	Respect for self-determination	No respect for self-determination
Japan (n = 41)^ab^	7	34
UK (n = 58)^a^	30	28
USA (n = 55)^b^	37	18

*p* = 8.0 × 10^−6^. Χ^2^ = 23.394. df = 2

^a^p = 0.003, Fisher’s exact test

^b^p = 3 × 10^−7^, Fisher’s exact test

### Factors that should be emphasized when assessing the mental capacity

Compared to Japan, the UK and the USA tended to place significantly higher importance on short-term memory, ability to express a choice or preference, ability to understand medical information given, ability to appreciate medical information as it relates to oneself, and ability to process reasonable information. Compared to the UK, the USA was significantly more likely to emphasize short-term memory, ability to understand medical information given, and ability to process information rationally. Compared to Japan and the US, the UK was significantly more likely to place importance on the ability to give appropriate weight to matters that are important to oneself (Table 5).

**Table 5:** Factor to be emphasized when assessing mental capacity of patients with anorexia nervosa

	Short-term memory	Ability to express a choice or preference	Level of psychopathological values	Ability to understand medical information given	Consciousness of disease	Ability to appreciate medical information as it relates to oneself	Ability to process reasonable information	Conscious level	Consistency of preference	Ability to weigh competing factors
Japan(n = 53)	5 (9%)^ab^	15 (27%)^de^	30 (55%)	49 (89%)^f^	35 (64%)	23 (42%)^hi^	24 (44%)^jk^	28 (51%)	16 (29%)	28 (51%)^m^
UK (n = 85)	49 (58%)^bc^	62 (73%)^e^	54 (64%)	75 (88%)^g^	60 (71%)	61 (72%)^i^	75 (88%)^kl^	53 (62%)	34 (40%)	75 (88%)^mn^
US (n = 85)	36( 42%)^ac^	54 (64%)^d^	54 (64%)	64 (75%)^fg^	63 (74%)	66 (78%)^h^	64 (75%)^jl^	48 (56%)	35 (41%)	52 (61%)^n^
P value	6.4 × 10^−8^ Χ^2^ = 33.1 df = 2	2.7 × 10^−7^ Χ^2^ = 30.2 df = 2	0.493 Χ^2^ = 1.4 df = 2	0.033 Χ^2^ = 6.8 df = 2	0.419 Χ^2^ = 1.8 df = 2	2.9 × 10^−5^ Χ^2^ = 20.9 df = 2	4.5 × 10^−8^ Χ^2^ = 33.8 df = 2	0.401 Χ^2^ = 1.8 df = 2	0.298 Χ^2^ = 2.4 df = 2	3.0 × 10^−6^ Χ^2^ = 25.5 df = 2

^a^p = 2.0 × 10^−6^ by Fisher’s direct test

^b^p = 2.17 × 10^−9^ by Fisher’s direct tes

^c^p = 0.033 by Fisher’s direct test

^d^p = 3.1 × 10^−5^ by Fisher’s direct test

^e^p = 1.23 × 10^−7^ by Fisher’s direct test

^f^p = 0.033 by by Fisher’s direct test

^g^p = 0.02 by Fisher’s direct test

^h^p = 2.8 × 10^−5^ by Fisher’s direct test

^i^p = 0.001 by Fisher’s direct test

^j^p = 2.9 × 10^−4^ by Fisher’s direct test

^k^p = 2.17 × 10^−8^ by Fisher’s direct test

^l^p = 0.046 by Fisher’s direct test

^m^p = 2.0 × 10^−6^ by Fisher’s direct test

^n^p = 7.8 × 10^−5^ by Fisher’s direct test

Compared to the USA and the UK, Japan showed a greater difference in trends of the factors emphasized when assessing the mental capacity. Therefore, after excluding Japan, we compared the factors considered to be of importance to physicians who evaluated patients with AN as having judgmental capacity and the factors considered to be of importance to physicians who evaluated patients with AN as having lost mental capacity. Physicians who rated patients with AN as competent to make decisions emphasized the following factors (in order of importance): ability to process reasonable information; ability to understand medical information given; ability to appreciate medical information as it relates to oneself; ability to express a choice or preference; and consciousness of disease. Physicians who rated patients with AN as lacking mental capacity emphasized the following factors (in order of importance): ability to understand medical information given; ability to weigh competing factors; ability to process reasonable information; level of psychopathological values; and consciousness of disease. Whether physicians rated the level of psychopathological values and ability to express a choice or preference as important resulted in a significant difference between the percentage of physicians who rated the patients as having the ability to make decisions and the percentage of physicians who rated the patients as not having the ability to make decisions (Table 6). The level of psychopathological values is the degree of values caused by AN (which can be changed by recovery) that affect the ability to think rationally (for example, distorted views of body weight).

**Table 6:** Factors to be emphasized when assessing mental capacity of patients with anorexia nervosa

	Short-term memory	Ability to express a choice or preference	Level of psychopathological values	Ability to understand medical information given	Consciousness of disease	Ability to appreciate medical information as it relates to oneself	Ability to process reasonable information	Conscious level	Consistency of preference	Ability to weigh competing factors
Full mental capacity (n = 113)	60/113 (53%)	83/113 (73%)	64/113 (57%)	93/113 (82%)	82/113 (73%)	88/113 (78%)	95/113 (84%)	67/113 (59%)	47/113 (42%)	81/113 (72%)
Impaired mental capacity (n = 57)	25/57 (44%)	33/57 (58%)	44/57 (77%)	46/57 (81%)	41/57 (72%)	39/57 (68%)	44/57 (77%)	34/57 (60%)	22/57 (39%)	46/57 (81%)
*p* value by Fisher’s direct method	0.165	0.031	0.006	0.476	0.534	0.125	0.187	0.549	0.418	0.137

## Discussion

This is the first study of how physicians treat and assess the mental capacity of patients with AN in Japan, the USA, and the UK. Patients with AN often refuse treatment, although this may seem irrational from a general perspective. Mental capacity is generally evaluated using four elements, understanding, appreciation, reasoning, and expression [3], and most patients with AN who refuse treatment have mental capacity when evaluated using these four elements [6, 15]. However, during this survey, approximately 20–30% of physicians in Japan, the UK, and USA rated the AN patient who refused treatment as having impaired mental capacity. Although it is possible that physicians are assessing the conventional four components of mental capacity of patients with AN refusing treatment as impaired, it is also possible that physicians treating patients with AN use other factors to assess mental capacity.

As a result of asking physicians whether they respect the patient’s decision to refuse treatment, approximately two-thirds of physicians in Japan, half of physicians in the UK, and one-third of physicians in the USA said that they do not. When patients with AN have mental capacity and refuse treatment, these physicians have higher regard for their ethical duty of beneficence to protect the patient's life than for their ethical duty of respect for autonomy (i.e., respecting the patient's self-determination). In particular, physicians in Japan showed significantly less respect for self-determination than those in the UK and the USA, which may be attributable to the fact that in Japan, in addition to long-standing paternalism [16, 17], there is a legal system that allows for therapeutic intervention for mentally ill patients as long as consent is received from the patient’s guardian [18]. It is possible that physicians in Japan have a different attitude toward the mental capacity of patients with AN who refuse treatment than physicians in the UK and the USA.

Physicians in Japan were significantly different from their counterparts in the USA and the UK in terms of which items they focus on when assessing the mental capacity of patients with AN. Physicians in Japan focus on short-term memory, ability to express a choice or preference, ability to understand medical information given, ability to appreciate medical information as it relates to oneself, ability to process reasonable information, and ability to weigh competing factors when assessing mental capacity. Of these, ability to express a choice or preference, ability to understand medical information given, ability to appreciate medical information as it relates to oneself, and ability to process reasonable information are components of the four mental capacity factors identified by Applebaum and Grisso [3]. Short-term memory is also an essential component of the ability to understand. The fact that these items were not emphasized by physicians in Japan when assessing mental capacity suggests that a standardized mental capacity assessment is not performed in Japan. One possible reason for this is that, in Japan, the distinction between the capacity to make clinical decisions and the capacity to be responsible during judicial psychiatric evaluations is not clearly defined [19], and physicians may not accurately understand the concept of mental capacity as the capacity to make decisions in clinical practice. A few clinicians may confuse the assessment of cognitive function with the assessment of the decision-making capacity in Japan [20]. The results showed that physicians in Japan might differ from those in the UK and the USA in terms of the evaluation methods used to determine mental capacity.

Physicians in the UK tended to place significantly more emphasis on short-term memory and ability to weigh competing factors when assessing the mental capacity than those in Japan and the USA. This may be because the Mental Capacity Act in the UK uses the following factors that refer to memory retention and the weighting of information to evaluate the mental capacity: understand the information relevant to the decision; retain that information; and use or weigh that information as part of the process of making decisions [13].

The difference in the emphasis placed on the factors of importance when assessing mental capacity was examined among physicians in the UK and the USA who reported that patients with AN who refuse treatment have mental capacity and those who reported that they do not have mental capacity. The results showed that physicians who thought the AN patient did not have mental capacity placed significantly more importance on level of psychopathological values than physicians who considered that the AN patient did have mental capacity; however, physicians who thought the AN patient had mental capacity placed significantly more importance on the ability to express a choice or preference than physicians who thought that the AN patient did not have mental capacity. No differences were found for other items.

Most items used to determine mental capacity are items from the aforementioned mental capacity assessment tool commonly used and items provided by the Mental Capacity Act [3]. No differences were found in the items normally used to assess mental capacity. Some physicians regarded some patients as having mental capacity and others did not despite the lack of differences in these items, which is consistent with the results reported by Elzakkers, who found that the concordance between the MacCAT-T results and the judgement of clinicians was not high [8].

The ethical attitude of physicians who value patient autonomy may lead to patients with AN being considered to have the mental capacity to make decisions whenever possible. As a result, physicians who assess mental capacity may place more weight on whether patients with AN express any preferences or choices.

Because patients with AN tend to be considered to have mental capacity when evaluated using the usual method of assessment [6, 15], when they refuse treatment, the physician is forced to choose between a paternalistic response that gives priority to treatment against the patient’s wishes in accordance with the duty of beneficence and a response that respects the patient’s self-determination and rejects treatment. However, by adding the level of psychopathological values to the evaluation items used to assess the presence or absence of mental capacity, it will be possible to consider patients who refuse treatment despite a life-threatening situation as having impaired mental capacity. This is because the refusal to treat patients with AN is related to the psychopathological value of not wanting to eat because of the fear of becoming obese, even if it means that the outcome will be death [21]. As a result, the ethical dilemma of whether to respect the patient’s self-determination or prioritize the protection of the patient’s life can be avoided. Tann et al. [6] pointed out the influence of beliefs and changes in the patient’s values on the mental capacity of patients with AN. Moreover, psychiatrists may be assessing the mental capacity of patients with AN in a different way [15], and the key factor involved in their assessment method may be the level of psychopathological values.

It may be necessary to add levels of psychopathological values to the assessment of mental capacity in relation to obesity fears and emotional disturbances of patients with AN. For example, patients with AN often experience severe anxiety when their weight increases, even if that increase is small, and they may refuse life-sustaining tube feeding and intravenous infusion because they contain fat or sugar. Elburg et al. [22] reported that the MacCAT-T places more emphasis on the cognitive aspects of decision-making, and that patients with AN often cannot control their emotions, which affect their decision-making ability; therefore, they often have the inability to make rational decisions. This emotional disturbance is typically related to a strong fear of treatment and anxiety about recovery, which result in weight gain, and losing one's identity [23], which is thought to be linked to body weight [24]. However, even if emotional disturbances resulting from the psychopathology affect the decision-making ability, it is unknown whether they can be added to the assessment of the mental capacity. The ability to make decisions is the basis of mental capacity. If patients have mental capacity, then they must be respected as an autonomous being; in contrast, if patients have impaired mental capacity, then they must be supported to become an autonomous being [25]. In other words, instead of disregarding the intentions of the patients by treating them as nonautonomous, they must be helped to make autonomous decisions by being provided with repeated explanations and appropriate concern for their feelings. If the patient has the mental capacity of an autonomous being, then the physician is ethically required to respect that patient's self-determination. This concept of autonomy is strongly influenced by Kantian philosophy and assumes that rational individuals can deliberate and govern their actions [26]. For example, when patients become desperate because of shock and fear resulting from discovering that they are ill and insist on refusing treatment even though that treatment will most likely result in a cure, it is not ethical to regard their insistence as self-determination and accept their decision according to the principle of respect for autonomy. This is because the principle of respect for autonomy involves an active duty of the physician, who has an obligation to assist the patient with decision-making in a rational and personable manner. In other words, when individuals make decisions based on emotions rather than rational thoughts, we believe that they are not fully exercising their autonomy. Therefore, when psychopathology causes emotional disorders, and these emotions strongly influence decision-making, such as what often occurs with patients with AN, they are considered to have lost their autonomy. Because mental capacity is related to patient autonomy, it is reasonable to consider patients with AN who have been severely affected by psychopathology to have impaired mental capacity.

This study had some limitations. First, the Japanese survey was mailed to physicians who were members of the Japanese Society for Eating Disorders, and the UK and USA surveys were web-based surveys of physicians who were members of academic societies and registered with medical networks. Although responses were obtained from physicians who treat patients with AN in all three countries, it is undeniable that the difference in survey methods may have affected the results. Second, the small sample size resulted in problematic representativeness. Third, the survey was a multiple-choice questionnaire that may have overlooked the possibility that physicians who treat patients with AN may place importance on mental capacity evaluation items that were not included as response options. Fourth, the use of the vignette for this study did not result in physicians making decisions based on a robust clinical assessment; instead, they made decisions based on an extremely limited understanding of the patient. The study results and differences observed may reflect idiosyncratic responses of the physicians to the case presented and not usual clinical practices, thus limiting the generalizability. Fifth, the vignette used in this study was simple and did not contain detailed clinical information. Therefore, each physician could have had different impressions of the case presented in the questionnaire, which may have influenced their answers. Sixth, the generalizability of these results is limited because the Japanese survey did not have a high response rate, and the response rates for the UK and USA surveys are unknown. Finally, because this was a vignette-based study, there were limitations to the interpretation of the results. For example, it is not possible to distinguish whether physicians chose coercive treatment as a result of their assessment of the lack of mental capacity when considering the psychopathological values of the patients, or whether their assessment of the lack of mental capacity was the result of considering psychopathological values in response to paternalistic acts. Qualitative research performed through interviews with physicians would be beneficial to address these issues.

Qualitative studies that perform interviews with physicians are needed to determine how physicians assess the mental capacity of patients with AN and how they make decisions when patients with AN refuse treatment. Additionally, further studies using several vignettes and different patient characteristics and more clinical details would provide a richer data set for analyses. Furthermore, it may be necessary to conduct a survey focused on which psychopathological values are important and why they are considered important.

## Conclusion

The results of mental capacity assessments of patients with AN performed by experienced physicians are inconsistent. In addition to the conventionally used items, the level of psychopathological values was found to be a key factor in the evaluation of mental capacity. Because the evaluation of mental capacity is an objective of the psychiatric evaluation, variations among the conclusions reached by physicians are undesirable. Patients with AN considered to have impaired mental capacity by psychiatrists have poorer treatment responsiveness [27]. The assessment of the mental capacity is also an important predictor of the treatment prognosis. Therefore, it is desirable to re-examine the method of evaluating the mental capacity to enable more effective assessments of the mental capacity of patients with AN.

## Supplementary Information

**Additional file 1.** Vignette Case and Question about factors of mental capacity.

## Data Availability

The data that support the findings of this study are available from the corresponding author on reasonable request.

## Abbreviations

AN: Anorexia nervosa
USA: United States
UK: United Kingdom
