# Supplementary material for: Key components of the mental capacity assessment of patients with anorexia nervosa: a study of three countries
Source: J Eat Disord. 2022 Jul 26;10:110. doi: 10.1186/s40337-022-00633-7 (PMC9327278; doi:10.1186/s40337-022-00633-7)
Supplement: Supplementary file 1 — Additional file 1. Vignette Case and Question about factors of mental capacity. [file 40337_2022_633_MOESM1_ESM.docx]

Appendix 1.

***Vignette Case: A patient with anorexia nervosa (AN) refusing treatment***

A patient with anorexia nervosa understands that they are considerably underweight and endangering their life by refusing treatment. They also still refuse hospitalization insisting that “I would rather be dead than gain weight, I do not want to be treated.” They seem to have no psychopathic symptoms, such as delusions or hallucinations.

***Question about factors of mental capacity***

Which factors do you consider when assessing the decision-making capacity of a patient with AN who is refusing treatment? (Multiple answer)

1. Short term memory

2. Ability to express a choice or preference

3. Level of psychopathological values, for example, distorted value regarding body weight

4. Ability to understand medical information given

5. Insight into disease

6. Ability to appreciate one’s medical condition after receiving explanation by a medical doctor

7. Ability to process reasonable information

8. Conscious level

9. Consistency of preference

10. Ability to weigh competing factors to reach the best choice regarding medical treatment
